# Biodiversity Promotes Tree Growth during Succession in Subtropical Forest

**DOI:** 10.1371/journal.pone.0081246

**Published:** 2013-11-26

**Authors:** Martin Barrufol, Bernhard Schmid, Helge Bruelheide, Xiulian Chi, Andrew Hector, Keping Ma, Stefan Michalski, Zhiyao Tang, Pascal A. Niklaus

**Affiliations:** 1 Institute of Evolutionary Biology and Environmental Studies, University of Zurich,Zürich, Switzerland; 2 Institute of Biology / Geobotany and Botanical Garden, Martin Luther University, Halle-Wittenberg, Halle (Saale), Germany; 3 Department of Ecology, College of Urban and Environmental Sciences, and Key Laboratory for Earth Surface Processes of Ministry of Education, Peking University, Beijing, China; 4 Institute of Botany, Chinese Academy of Sciences, Beijing, China; 5 Department of Community Ecology, Helmholtz Centre for Environmental Research, UFZ, Halle (Saale), Germany; University of Marburg, Germany

## Abstract

Losses of plant species diversity can affect ecosystem functioning, with decreased primary productivity being the most frequently reported effect in experimental plant assemblages, including tree plantations. Less is known about the role of biodiversity in natural ecosystems, including forests, despite their importance for global biogeochemical cycling and climate. In general, experimental manipulations of tree diversity will take decades to yield final results. To date, biodiversity effects in natural forests therefore have only been reported from sample surveys or meta-analyses with plots not initially selected for diversity. We studied biomass and growth of subtropical forests stands in southeastern China. Taking advantage of variation in species recruitment during secondary succession, we adopted a comparative study design selecting forest plots to span a gradient in species richness. We repeatedly censored the stem diameter of two tree size cohorts, comprising 93 species belonging to 57 genera and 33 families. Tree size and growth were analyzed in dependence of species richness, the functional diversity of growth-related traits, and phylogenetic diversity, using both general linear and structural equation modeling. Successional age covaried with diversity, but differently so in the two size cohorts. Plot-level stem basal area and growth were positively related with species richness, while growth was negatively related to successional age. The productivity increase in species-rich, functionally and phylogenetically diverse plots was driven by both larger mean sizes and larger numbers of trees. The biodiversity effects we report exceed those from experimental studies, sample surveys and meta-analyses, suggesting that subtropical tree diversity is an important driver of forest productivity and re-growth after disturbance that supports the provision of ecological services by these ecosystems.

## Introduction

Tropical and subtropical forests range among the most productive ecosystems on Earth [1,2], significantly interacting with global biogeochemical cycles and regulating climate at the regional and global scale [3,4]. These ecosystems harbor a significant fraction of global plant diversity and are under increasing pressure from land-use change and intensification, associated fragmentation, as well as climate change [5] and pollution [6]. 

Experimental and theoretical evidence demonstrates that the functioning of many ecosystems depends on the diversity of their component species [7,8]. To date, most studies manipulating plant species diversity have been conducted in artificially established herbaceous communities, mostly in temperate climates, with comparably limited information on biodiversity-ecosystem functioning relationships in tree communities. Nevertheless, a number of recent survey studies and meta-analyses confirmed a generally positive correlation between species richness and primary productivity also in forests. Evidence bases on data originating from forest plantations and on survey studies in forest inventory plots. The plantations for which data are available often are characterized by the dominance of commercially important, fast-growing species, low stand ages and a lower structural complexity than the one found in natural or semi-natural forests. Therefore, these two study types are complementary and may well reveal different biodiversity-productivity relationships. 

Using meta-analysis, Piotto [9] analyzed an extensive data set compiled from a large number of tropical and temperate plantations. Tree species mixtures had higher productivity than monocultures, but no significant effect of tree species richness within mixtures was found. Vilà et al. [10] analyzed data from European forest plots differing in forest type and found a 24% increase in productivity in mixtures relative to monospecific stands. Both studies suggest that the productivity increase with diversity saturates at relatively low diversity levels. Paquette & Messier [11] studied productivity in forest inventory plots spanning a gradient from temperate to boreal, and found that diversity was an important determinant of productivity in boreal but not in temperate forests. Common to these studies, and complicating data interpretation, is the need to control for effects of drivers of tree diversity co-varying with species richness, in particular climate, soil fertility, and successional age, which is not always done [11]. Most sample surveys in natural and semi-natural forest were carried out in temperate or boreal forests characterized by rather low species diversity. For example, tree species richness averaged around two in the survey by Vilà et al. [10], with 51% of the plots being monocultures and another 42% being two or three species mixtures. The sample survey by Paquette & Messier [11] consisted of slightly more diverse plots (average of 3–5 species, depending on biome). An important conclusion emerging from the available literature is that data for natural subtropical and tropical forests is largely underrepresented or missing, despite their high species diversity and the importance of these ecosystems for the regulation of global processes.

Here, we adopted a comparative study design, deliberately selecting plots in a highly diverse subtropical secondary forest in south-east China to represent different levels of tree species richness and successional age. The comparative study approach has a higher power to detect effects of diversity, and is more likely to reveal causal relationships than sample surveys, but surprisingly has never been used to assess biodiversity–productivity relationships in forests. As dependent variables we measured the basal area and the two-year increase in basal area of all trees in the study plots. While effects of tree species richness are ultimately caused by functional differences among the species present in a community, this trait variation may not be fully captured by species numbers. We therefore also analyzed our data in relation to the diversity of a range of growth-related functional traits. To account for additional functional trait variation possibly reflected in phylogeny, we further included an index of phylogenetic diversity in our analyses. In this study, we successfully (1) tested for effects of tree species richness on productivity (2), compared the effect sizes we found to findings from other studies in forest but also grassland, and (3) tested whether functional or phylogenetic diversity would explain variation in the observed responses that is not explained by species richness.

## Methods

### Study site and experimental design

Field plots selected to represent different levels of tree species richness and successional age were established in Gutianshan National Nature Reserve, western Zhejian province, China (29°15’ N, 118°07’ E; mean annual temperature: 15.1°C, maximum: 38.1°C in July; mean annual precipitation: ~2000 mm; permission for this field study was granted by the Administration Bureau of the Gutianshan National Natural Reserve, Kaihua, China). Prior to its establishment in 1975, the ~81 km^2^ site was managed as commercial forest planted with *Pinus massoniana* and *Cunninghamia lanceolata* [12]. Today, 1462 seed plant species belonging to 684 genera and 149 families are found in the reserve. The >250 tree species present include members with temperate (e.g. Fagaceae), subtropical (e.g. Anacardiaceae, Lauraceae) and tropical (e.g. Symplocaceae, Theaceae, Myrsinaceae) distribution, resulting in a diversity similar to the one of tropical forests [13,14].

Twenty-seven plots of 30 × 30 m area were deliberately selected to span factorial gradients in both tree species richness and successional age resulting from timber cutting by local communities. Average distance between plot pairs was ~3 km. The closest pair was 40 m apart, followed by 165 m and 243 m for the next-closest pairs. For each plot, we determined tree species richness from the inventory data we recorded (see below). Successional age was assigned to five age classes (<20, 20–40, 40–60, 60–80, or >80 years old) based on the age of the fifth-largest tree of each plot (determined from a stem core), because the precise date of the last logging event could generally not be determined. Our goal was to evenly cover the range in tree diversity and successional ages present at the site, although it was not possible to keep these two fixed, independent variables fully orthogonal to each other. In the further course of the study, two plots were lost due to (illegal) timber cutting. All analysis presented are therefore based on data from the remaining twenty-five plots.

We did not select plots randomly, because such a “sample survey” design would have resulted in a concentration of plots around mean tree species richness values, with a typically bell-shaped distribution. In sample surveys (and meta-analyses based on sample surveys), correlations between species richness and productivity are bi-directional relationships between two dependent variables. This problem can be alleviated by fixing one variable as independent variable at different levels that are similarly replicated, and then measuring the other variable as dependent variable. This approach is recommended e.g. in the classical statistical textbook by Snedecor & Cochran [15] who refer to this type of study as *comparative study* and rank it between *sample surveys* and *designed experiments* (with randomized treatments) with regard to the power to detect causal relationships between variables. 

### Tree size and growth

We tagged all tree individuals with a diameter at breast height of at least ten centimeters. The resulting “canopy” tree cohort comprised of 1523 trees belonging to 66 species, 49 genera, and 29 families (Table S1 in File S1). In the central 10 × 10 m quadrat of each plot, all trees with a diameter of at least three but less than ten centimeters were also tagged. This “understory” tree cohort consisted of 672 individuals belonging to 58 species, 34 genera, and 19 families (Table S2 in File S1).

The diameter at breast height of all “canopy” and “understory” trees was determined in summer 2008 and again in 2010, using either permanently installed dendrometer bands or a metering tape. Tree diameters were converted into stem cross-sectional area (basal area). We further calculated basal area increments from 2008 to 2010 as a proxy of tree growth.

### Functional diversity

We determined functional diversity sensu Petchey & Gaston [16], using a range of potentially growth-related species traits. For all species present in either the “canopy” or “understory” cohort, we recorded leaf seasonality (evergreen vs. deciduous), leaf habit (broadleaved vs. coniferous), specific leaf area (SLA), leaf carbon to nitrogen ratio (C:N), leaf size (dry weight of a typical mature leaf), the typical maximum height reached by mature individuals of the species, and the typical density of stem wood. Data were generally recorded on individuals sampled in Gutianshan National Nature Reserve [17,18]. For ten species, wood density was taken from the global wood density data base [19], re-scaling values based on the correlation of wood densities of species present in both data sets. All traits were normalized to zero mean and unit variance; twenty-one out of 282 values for leaf size, C:N, and SLA were missing and set to zero. Functional diversity was then calculated as total branch length of the functional-trait dendrogram (euclidian distances, complete linkage agglomeration), calculated for the particular set of species occurring in a plot (Fig. S1 in File S1).

### Phylogenetic diversity

Phylogenetic diversity was calculated based on sequence information (*matK*, *rbcL* and the ITS region including the 5.8s gene) retrieved from GenBank or obtained using standard barcoding protocols. In brief, a phylogenetic tree including 440 woody species present at the field site was generated using a maximum likelihood (ML) method. Using the ML topology and branch lengths, an ultrametric tree was created by non-parametric rate smoothing, with 27 node ages constrained by published fossils and a fixed age of 125 million years for the crown node of the Eudicots. Data processing and the construction of the phylogenetic tree are reported in detail in Method S1 in File S1. Phylogenetic diversity was calculated as total branch length defined by the subset of species occurring in a plot (see Fig. S2 in File S1).

### Evenness

Although plots were selected for species richness, we calculated species evenness based on the number of stems recorded for each species. We chose the evenness index E_1/D_=1/(DS), where D is Simpson’s index of dominance and S species richness. We preferred E_1/D_ over Shannon-Wiener-based indices because E_1/D_ is independent of S [20].

### Statistical analysis

Effects of tree diversity and successional age were tested by fitting multiple regression models with sequential sum of squares (lm function of R 2.15.0; http://www.r-project.org). Species richness effects were tested either independently of successional age (richness fitted before age), or after adjusting for effects of age (richness fitted after age). Since species richness was analyzed as continuous variable, effect sizes need to be reported based on an arbitrarily chosen change in species richness. Here, we report predicted changes in the analyzed variable for a hypothetical increases in species richness from ten to twenty species.

Plots had originally been selected by visually choosing plots with low, intermediate, or high diversity. The exact numbers of species was determined later. We therefore also repeated our analysis by fitting models with diversity as tree-level ordinal factor (each level containing approximately 1/3 of the plots), which better reflects the original plot selection procedure. Both analyses resulted in very similar results; we therefore only report data from the analyses including exact species richness.

Influences of site conditions were tested using the covariates elevation, slope aspect (north-south and east-west component), slope inclination, soil pH, soil moisture, and soil organic C and N. Since covariables often are collinear and will always explain some variation in the data set, even if just by chance, we normalized them (scaling to zero mean and unit variance) and aggregated these to orthogonal principle components. We then tested for effects of the (orthogonal) first two principal components by including these as covariates in our linear models.

We tested whether the number of trees found in the second census but not the first depended on species richness or successional age, i.e. whether non-random ingrowth of individuals into the cohort assessed occurred. Similarly, we tested for effects on the number of trees lost from the assessed size classes, i.e. whether non-random mortality or transitions from the understory to the canopy tree cohort occurred. These tests were conducted by fitting generalized linear models with log-link and Poisson error model accounting for overdispersion, if necessary (glm function of R). 

Structural equation models including effects of tree diversity (latent variable defined by tree species richness, functional diversity, and phylogenetic diversity), successional age, and their indirect effects mediated by changes in tree density (i.e. the number of trees per plot) were fitted by generalized least squares (sem function, http://cran.r-project.org/web/packages/lavaan). Tree density was included as intermediate explanatory variable, because positive effects of biodiversity on plot-level cumulated size measures must, as a mathematical necessity, result from increases in the size of individuals, increases in their numbers, or from both. 

## Results

### Richness effects on tree size and growth

**Figure 1:**
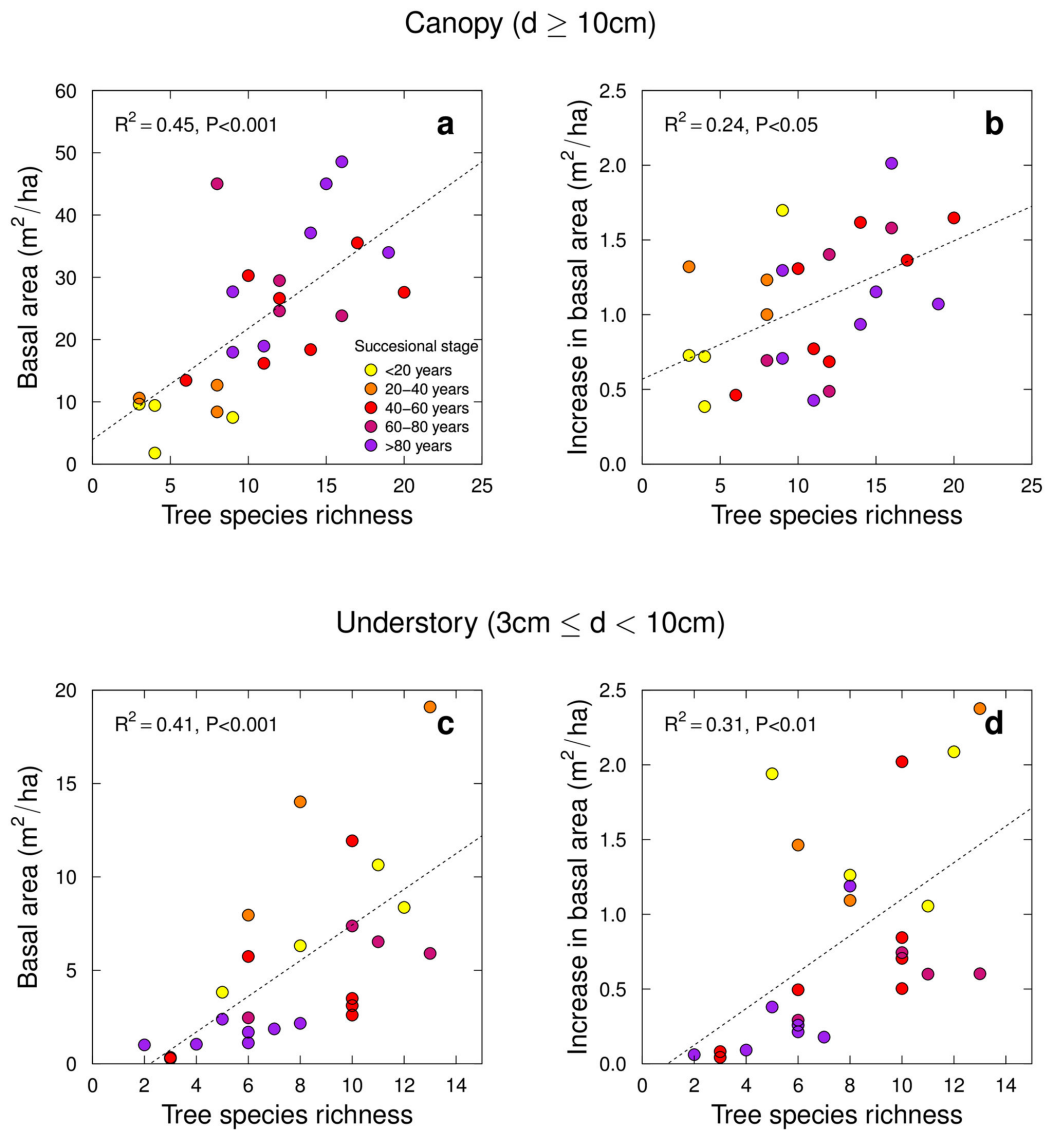
Total stem basal area in 2008 (a, c) and increment of total stem basal area from 2008 to 2010 (b, d) as functions of tree species richness and successional age of the study plots. Growth was assessed separately for canopy trees with a diameter at breast height (d) of 10 cm or larger (a, b) and for understory trees with d between 3 and 10 cm (c, d).

In the “canopy” cohort (trees with diameter ≥ 10cm), total stem basal area per plot in 2008 increased linearly with tree species richness, which explained 45% of the observed variation (F_1,22_=26.9, P<0.001 in multiple regression with sequential sum of squares for richness followed by successional age; Figure 1a). Similarly, tree species richness explained a significant fraction of variation in the 2008–2010 increment in total stem basal area, a proxy for stand growth (F_1,22_=7.7, P=0.01 in multiple regression with richness followed by successional age; Figure 1b). Per 10 extra species, these effects correspond to an additional stem basal area of 17.8±4.1 m^2^/ha, or a +82% increase when doubling species number from 10 to 20. For the 2008–2010 growth of stem basal area, these numbers correspond to 0.46±0.17 m^2^/ha, equivalent to a +45% change when increasing tree species number from 10 to 20. Despite the partial confounding of measured tree species richness and successional age (Pearson’s product moment correlation, r=0.60, P<0.01), effects of tree species richness remained significant after adjusting for successional age (F_1,22_=4.5, P=0.04 for total stem basal area; F_1,22_=9.8, P<0.01 for increment of total stem basal area; richness fitted after successional age in multiple regression). These adjusted effects correspond to a +44% increase in stem basal area and a +62% increase in stem basal area increment when increasing species numbers from 10 to 20.

The same positive relationship between tree species richness and dependent variables was also found for “understory” trees (3cm ≤ diameter < 10cm; Figures 1c, d). In contrast to the “canopy” tree cohort, tree species richness and successional age were marginally significantly negatively correlated for understory trees (r=–0.34, P=0.1). Species richness of understory trees explained a significant fraction of their total stem basal area in 2008 (F_1,22_=20.2, P<0.001) and their increment in total stem basal area from 2008–2010 (F_1,22_=1.4, P<0.001). These effects correspond to an increase in basal area of 9.55±2.37 m^2^/ha (+129%) and an increase in basal area growth of 1.22±0.38 m^2^/ha (+111%) when doubling understory species numbers from 10 to 20. These effects remained significant (F_1,22_=11.3, P<0.01 and F_1,22_=6.3, P=0.02) when first adjusting for successional age and correspond to a +103% increase in stem basal area and a +75% increase in stem basal area growth when doubling understory species numbers from 10 to 20.

In our study, species richness and evenness were significantly negatively correlated, both for the canopy (r=-0.5, P=0.01) and the understory tree cohort (r=-0.57, P=0.003). As a consequence, basal area and basal area increment were significantly negatively related to evenness for both size cohorts (P=0.04 for canopy tree basal area increase, P<0.001 for all other cases).

The wood density of the species recorded in our census spanned a factor of approximately two. If substantial shifts in wood density of the species present would occur with diversity, this would bias basal area as an indicator of productivity. To explore this possibility, we repeated our multiple regression analysis, this time scaling basal area with the wood density of the species. Results remained essentially the same and are therefore not reported here. Note, however, that this analysis is not properly replicated since wood density was not determined on a per-plot basis but considered a constant property of the species, which clearly is simplistic.

When covariates describing plot characteristics were included in the analysis (aggregated as principle components), effects of species richness remained significant in all cases. These covariates explained virtually no variance in the data (on average less than 1%) except for basal area of the canopy cohort (7% and 17%).

### Functional diversity, phylogenetic diversity, and stand density

**Figure 2:**
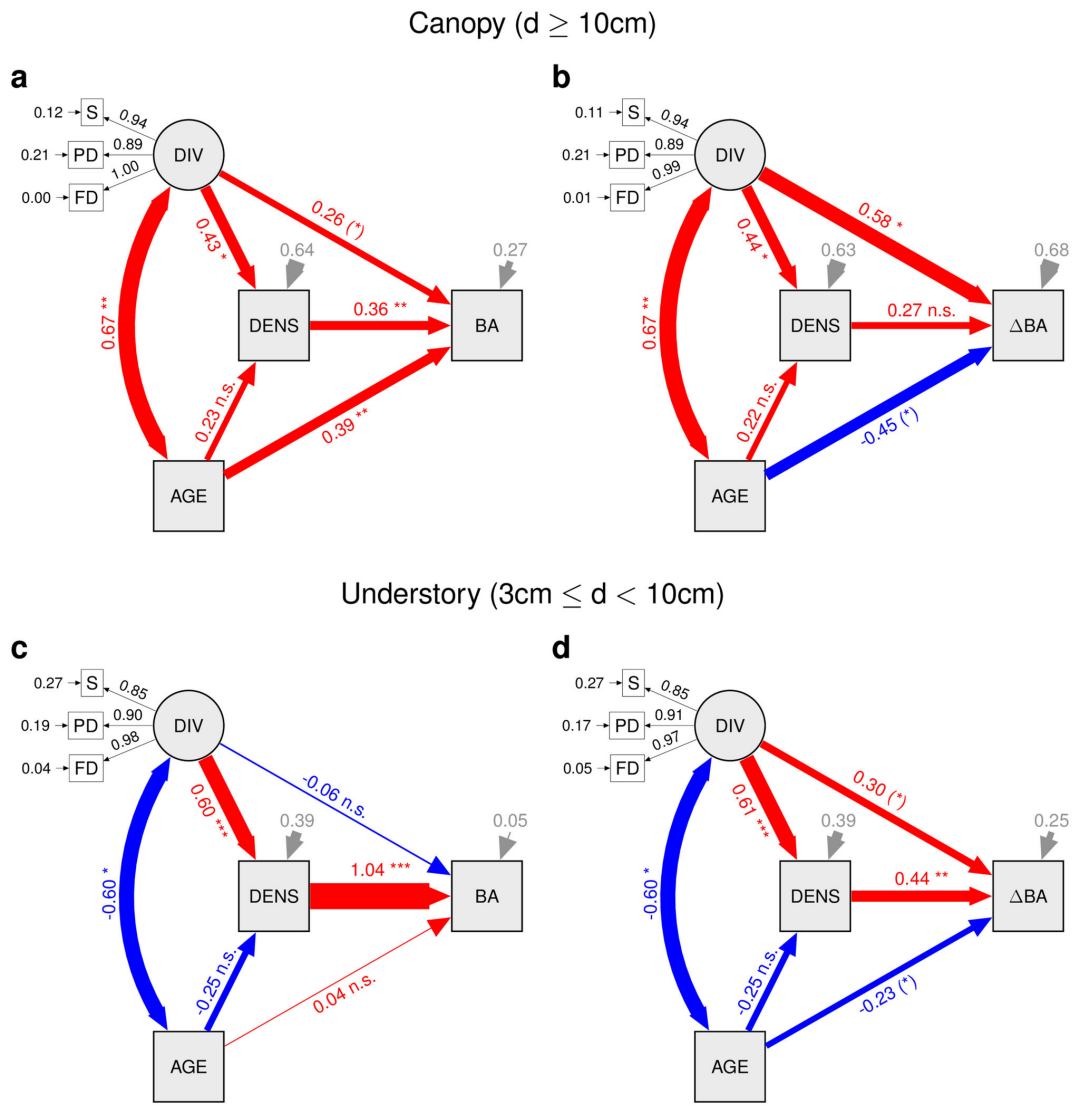
Structural equation models fitting total stem basal area in 2008 (a, c) and increment of total stem basal area from 2008 to 2010 (b, d) in dependence of successional age, tree diversity, and tree stem density. Path diagrams indicate effects of tree species diversity on the two dependent variables, either directly or indirectly via increases in tree density. The diagrams show standardized path coefficients (red: positive; blue: negative) and associated statistical significances (*** P<0.001; ** P<0.01; * P<0.05; (*) P<0.1). Variable abbreviations: S = species richness, PD = phylogenetic diversity, FD = functional diversity, DIV = diversity (latent variable related to previous three), AGE = successional age, DENS = tree density, BA = total stem basal area, ∆BA = increment of total stem basal area.

Structural equation models including diversity as a latent variable combining species richness, functional diversity, and phylogenetic diversity indicated that successionally older stands had higher total stem basal area and reduced growth of canopy trees (Figure 2a, b; path coefficients linking AGE with BA and ∆BA, respectively). In contrast, diversity increased total stem basal area (Figure 2a), with a substantial component of the effect being indirect via increases in the number of trees, i.e. in stand density. Diversity also increased total stem basal area growth (Figure 2b). 

Successional age exerted little influence on understory trees. However, diversity also increased basal area and basal area increments in this cohort, with effects being mediated primarily by increases in number of trees per plot rather than by enhanced individual growth (Figure 2c, d). 

**Figure 3:**
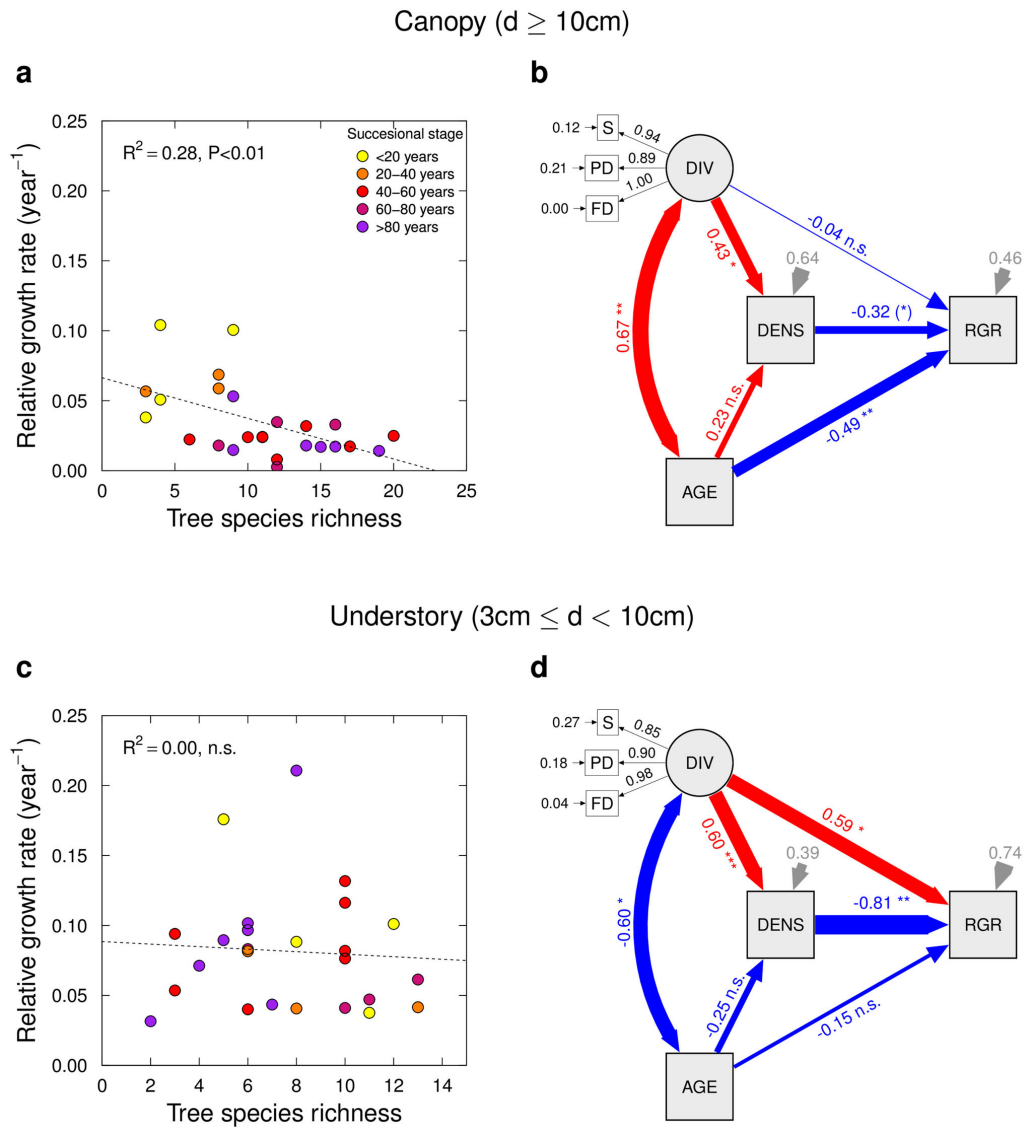
Relative growth rate of individual stem basal area (RGR, 2008–2010 period) in dependence of successional age, tree species richness, and tree stem density. In the canopy tree (d>10 cm) cohort, RGR declines with diversity (a) due to its correlation with successional age (b; path from DIV via AGE to RGR); in the understory (3 cm<d<10 cm) cohort, a positive direct and negative indirect (via density) effect of species richness on RGR balance each other out (c, d). The diagram shows standardized path coefficients (red: positive; blue: negative) and associated statistical significances (*** P<0.001; ** P<0.01; * P<0.05; (*) P<0.1). Variable abbreviations: S = species richness, PD = phylogenetic diversity, FD = functional diversity, DIV = diversity (latent variable related to previous three), AGE = successional age, DENS = tree density, BA = total stem basal area, ∆BA = increment of total stem basal area.

Relative growth rates of individual trees decreased in the canopy cohort (P<0.01, linear model, Figure 3a). Structural equation modeling suggested that this effect was driven indirectly by increased number of trees at high diversity (i.e. increased stand density), which resulted in reduced individual growth rates (Figure 3b). In the understory cohort, positive effects of diversity on individual growth rates were cancelled by negative effect via increased density, resulting in the absence of an overall effect (Figure 3c,d).

### Transitions between size cohorts

Using general linear models with binomial error distribution, no effects of species richness or successional age on the in-growth into the canopy tree cohort were found. Similarly, we did not record statistically significant losses from the censored cohorts. Also, the number of additional trees found in the second census and the number of trees lost between censuses was small (8.5 and 2.3 individuals per plot, respectively, in the canopy cohort; corresponding numbers were 1.7 and 1.4 in the understory cohort).

## Discussion

Our results indicate strong, positive effects of tree species richness on stand total basal area and growth. These effects were primarily mediated by increased individual growth of the bigger trees reaching the canopy, and by an increased density of individuals in the smaller understory trees. Functional and phylogenetic diversity were strongly positively correlated to species richness, and did not explain substantial variation in addition to the effects explained by species numbers.

Stand growth as assessed by increment of total stem basal area can underestimate woody biomass accumulation due to allometric scaling. It is therefore even more remarkable that the diversity effects we report here are larger than effects reported for many herbaceous and woody ecosystems. Normalized effect sizes Zr ranged from 0.44–0.81 for total stem basal area and from 0.51–0.63 for increment of total stem basal area in our study, depending on whether effects of successional age were adjusted prior to testing the effect of species richness and whether the canopy or understory tree cohort was considered; these effect sizes appear exceptionally high when compared to a recent meta-analysis [8,21] in which only few field studies showed similar or higher Zr for primary production; these generally were the studies in which biodiversity effects were strongly driven by legume responses [22]. The available meta-analysis comparing forest polycultures to monocultures also report much lower effects; for example, Piotto [9] reports a cumulated effect size of d+=0.31, corresponding to Zr≈0.32, and Zhang et al. [23] reports a 24% higher productivity in mixtures, with a saturation of the productivity at a diversity of about six species. Our findings are thus in line with recent reports [24] indicating that biodiversity contributions to ecosystem change are similarly important as effects of other global change drivers, including eutrophication and atmospheric CO_2_ increases.

Our findings contrast with the view that biodiversity–ecosystem functioning relationships are weaker in natural than in experimental systems [8,25], or even absent due to high species similarity resulting from strong environmental filtering [26]. The positive relation between richness and productivity we report is opposite to a trend often observed in sample surveys along environmental gradients [15] in which high productivity coincides with low species diversity [27,28]. Such a negative correlation between biodiversity and productivity has often been attributed to competitive exclusion under increased productivity, and to dissimilarities in local species pool composition, i.e. β-diversity [29]. In our study, the strong positive correlation between biodiversity and productivity indicates that these factors were not at play at the spatial scale covered, or that these were of subordinate importance. Indeed, it is conceivable that biodiversity effects are even stronger in natural communities than in randomly assembled experimental communities [30], potentially due to pronounced species differences resulting from processes limiting similarity (e.g. competition for the same ressources, or effects of shared pathogens) [31,32].

Interestingly, density-mediated biodiversity effects on growth have also been found in an experimental study with herbaceous communities [33], with individual sizes orders of magnitude lower than for trees. The larger number of individuals we found in more diverse plots possibly results from complementarity among species, i.e. from reduced competition between individuals due to niche differentiation and facilitation. Similar effects have been found in experimental herbaceous communities [33]. While the relationship between density and diversity is potentially bi-directional, structural equation models that do not allow effects of diversity mediated via density (and which therefore may be overly conservative) largely confirmed our findings (Figure S3 in File S1).

Species richness and evenness were significantly negatively correlated in our experimental plots. Such a relation has also been found in designed experiments with artificially established herbaceous systems [34]. This inverse relation between richness and evenness is at least in part the result of the typical rank-abundance relationships found in natural communities, i.e. individual numbers decline rapidly with rank, leaving only few dominant and subdominant species. Under these conditions, increasing diversity essentially results in the addition of rare species, and evenness therefore declines. 

Controlling for factors other than diversity is crucial in non-experimental studies to rule out confounding with these other drivers. Plant species richness in subtropical forest communities (α-diversity) is determined by many factors, including habitat properties. In a 24 ha permanent forest plot nearby our study site, spatial structuring of habitat accounted for approximately one quarter of the variation in diversity between subplots (β-diversity) [13]. Nevertheless, at our study site the measured topographic and soil variables did not explain the observed diversity effects; also, a previous study of community structure in the same plots had shown that these covariates were unrelated to tree and shrub species richness [12]. Topography in Gutianshan Reserve is very rugged, evidenced in a broad variation in slope among our experimental plots; specific habitat properties may therefore scale with horizontally-projected plot area rather than with surface area. However, when we repeated the analyses using projected plot area, we obtained virtually identical results as when using surface area as reference.

Overall, our study suggests that tree species richness is an important factor enhancing community-level regrowth during secondary succession, contributing to resilience after disturbance. Our results further indicate that high tree species richness in these forests contributes to sustained growth even in old stands, either directly or indirectly through increased stand densities. Faster regrowth at high diversity may have important implications for a range of ecosystem services including erosion control and carbon storage. The rugged terrain at the field site, combined with high-intensity precipitation, renders slopes particularly susceptible to erosion and deterioration. Atmospheric source-sink balancing indicates a partly unresolved residual terrestrial CO_2_ sink [35]. Recent evidence indicates that secondary vegetation regrowth after wood harvesting contributes to this sink [36]. Our results hint at the possibility that the strength of this sink might depend on the biodiversity of forest stands.

## Supporting Information

File S1 (PDF)
