# Correction: Biodiversity Promotes Tree Growth during Succession in Subtropical Forest

**DOI:** 10.1371/annotation/48869dee-d4a9-4ff5-a401-ce73440e7ecf

**Published:** 2013-12-31

**Authors:** Martin Baruffol, Bernhard Schmid, Helge Bruelheide, Xiulian Chi, Andrew Hector, Keping Ma, Stefan Michalski, Zhiyao Tang, Pascal A. Niklaus

The name of the first author is spelled incorrectly. The correct name is: Martin Baruffol. 

The correct Citation is: Baruffol M, Schmid B, Bruelheide H, Chi X, Hector A, et al. (2013) Biodiversity Promotes Tree Growth during Succession in Subtropical Forest. PLoS ONE 8(11): e81246. doi:10.1371/journal.pone.0081246.

