# Supplementary material for: Biodiversity Promotes Tree Growth during Succession in Subtropical Forest
Source: PLoS One. 2013 Nov 26;8(11):e81246. doi: 10.1371/journal.pone.0081246 (PMC3841117; doi:10.1371/journal.pone.0081246)
Supplement: File S1 — (PDF) [file pone.0081246.s001.pdf]

Table S1: Family, genus, and species names of individuals in the canopy tree (d>10 cm) cohort. The number of individuals listed refers to all 25 study plots, i.e. to a total ground area of 22’500 m^2.

**Family**	**Genus**	**Species**	**Individuals**
Aceraceae	Acer	*amplum*	1
Aceraceae	Acer	*cordatum*	1
Alangiaceae	Alangium	*kurzii*	3
Anacardiacee	Toxicodendron	*succedaneum*	13
Aquifoliaceae	Ilex	*chinensis*	7
Aquifoliaceae	Ilex	*micrococca*	3
Aquifoliaceae	Ilex	*rotunda*	1
Araliaceae	Dendropanax	*dentiger*	3
Betulaceae	Carpinus	*viminea*	3
Daphniphyllaceae	Daphniphyllum	*oldhamii*	69
Ebenaceae	Diospyros	*glaucifolia*	7
Elaeocarpaceae	Elaeocarpus	*chinensis*	4
Elaeocarpaceae	Elaeocarpus	*decipiens*	6
Elaeocarpaceae	Elaeocarpus	*japonicus*	1
Ericaceae	Rhododendron	*latouchea*	2
Ericaceae	Rhododendron	*ovatum*	1
Ericaceae	Vaccinium	*carlesii*	1
Euphorbiaceae	Sapium	*japonicum*	1
Fabaceae	Albizia	*kalkora*	17
Fabaceae	Dalbergia	*hupeana*	5
Fagaceae	Castanea	*henryi*	20
Fagaceae	Castanopsis	*carlesii*	26
Fagaceae	Castanopsis	*eyrei*	354
Fagaceae	Castanopsis	*fargesii*	43
Fagaceae	Castanopsis	*sclerophylla*	14
Fagaceae	Castanopsis	*tibetana*	3
Fagaceae	Cyclobalanopsis	*glauca*	18
Fagaceae	Cyclobalanopsis	*myrsinaefolia*	37
Fagaceae	Cyclobalanopsis	*stewardiana*	4
Fagaceae	Lithocarpus	*glaber*	89
Fagaceae	Quercus	*phillyreoides*	6
Fagaceae	Quercus	*serrata*	49
Hamamelidaceae	Liquidambar	*formosana*	4
Hamamelidaceae	Loropetalum	*chinense*	10

(continued next page)

(Table S1, continued)

Lauraceae	Cinnamomum	*subavenum*	2
Lauraceae	Litsea	*coreana*	1
Lauraceae	Machilus	*leptophylla*	1
Lauraceae	Machilus	*thunbergii*	24
Lauraceae	Neolitsea	*aurata*	2
Lauraceae	Sassafras	*tzumu*	1
Myricaceae	Myrica	*rubra*	40
Myrtaceae	Syzygium	*buxifolium*	2
Nyssaceae	Nyssa	*sinensis*	3
Oleaceae	Fraxinus	*insularis*	4
Oleaceae	Osmanthus	*cooperi*	1
Pinaceae	Pinus	*massoniana*	210
Pinaceae	Pinus	*taiwanensis*	34
Rosaceae	Laurocerasus	*spinulosa*	1
Rosaceae	Malus	*leiocalyca*	1
Rosaceae	Photinia	*beauverdiana*	1
Rosaceae	Prunus	*schneideriana*	2
Rosaceae	Sorbus	*dunnii*	1
Sabiaceae	Meliosma	*flexuosa*	3
Sabiaceae	Meliosma	*oldhamii*	7
Schoepfiaceae	Schoephia	*jasminodora*	1
Styracaceae	Alniphyllum	*fortunei*	40
Styracaceae	Styrax	*dasyanthus*	1
Styracaceae	Styrax	*odoratissimus*	1
Styracaceae	Styrax	*suberrifolia*	1
Symplocaceae	Symplocos	*stellaris*	1
Taxodiaceae	Cunninghamia	*lanceolata*	11
Theaceae	Adinandra	*millettii*	8
Theaceae	Schima	*superba*	284
Theaceae	Ternstroemia	*gymnanthera*	5
Tiliaceae	Tilia	*endochrysea*	2
Ulmaceae	Celtis	*biondii*	1

Table S2: Family, genus, and species names of individuals in the understory tree (3 cm<d<10 cm) cohort. The number of individuals listed refers to the central 10 × 10 cm quadrat of all 25 study plots, i.e. to a total ground area of 2500 m^2.

Family	Genus	Species	Individuals
Anacardiaceae	Rhus	*hypoleuca*	4
Anacardiacee	Toxicodendron	*succedaneum*	8
Aquifoliaceae	Ilex	*buergeri*	4
Aquifoliaceae	Ilex	*chinensis*	5
Aquifoliaceae	Ilex	*litseifolia*	10
Aquifoliaceae	Ilex	*pubescens*	1
Aquifoliaceae	Ilex	*suaveolens*	1
Aquifoliaceae	Ilex	*wilsonii*	1
Daphniphyllaceae	Daphniphyllum	*oldhamii*	9
Ericaceae	Lyonia	*ovalifolia*	2
Ericaceae	Rhododendron	*latouchea*	27
Ericaceae	Rhododendron	*mariesii*	2
Ericaceae	Rhododendron	*ovatum*	26
Ericaceae	Vaccinium	*bracteatum*	6
Ericaceae	Vaccinium	*carlesii*	7
Ericaceae	Vaccinium	*mandarinorum*	3
Fabaceae	Albizia	*kalkora*	5
Fagaceae	Castanopsis	*carlesii*	4
Fagaceae	Castanopsis	*eyrei*	44
Fagaceae	Castanopsis	*fargesii*	6
Fagaceae	Castanopsis	*sclerophylla*	12
Fagaceae	Castanopsis	*tibetana*	2
Fagaceae	Cyclobalanopsis	*glauca*	31
Fagaceae	Cyclobalanopsis	*myrsinaefolia*	6
Fagaceae	Cyclobalanopsis	*stewardiana*	34
Fagaceae	Lithocarpus	*glaber*	91
Fagaceae	Quercus	*serrata*	29
Hamamelidaceae	Corylopsis	*glandulifera*	15
Hamamelidaceae	Liquidambar	*formosana*	2
Hamamelidaceae	Loropetalum	*chinense*	13
Lauraceae	Cinnamomum	*subavenum*	4
Lauraceae	Litsea	*coreana*	1
Lauraceae	Litsea	*elongata*	1
Lauraceae	Machilus	*thunbergii*	11
Myricaceae	Myrica	*rubra*	8
Myrtaceae	Syzygium	*buxifolium*	13
Pinaceae	Pinus	*massoniana*	16
Pinaceae	Pinus	*taiwanensis*	1

(continued next page)

(Table S2, continued)

Rosaceae	Photinia	*glabra*	1
Rosaceae	Photinia	*parvifolia*	2
Rosaceae	Sorbus	*folgneri*	4
Rubiaceae	Randia	*cochinchinensis*	2
Sabiaceae	Meliosma	*flexuosa*	1
Sabiaceae	Meliosma	*oldhamii*	1
Saxifragaceae	Itea	*chinensis*	6
Styracaceae	Styrax	*odoratissimus*	5
Symplocaceae	Symplocos	*anomala*	5
Symplocaceae	Symplocos	*setchuensis*	1
Symplocaceae	Symplocos	*stellaris*	4
Symplocaceae	Symplocos	*sumuntia*	8
Theaceae	Adinandra	*millettii*	18
Theaceae	Camellia	*chekiang-oleosa*	12
Theaceae	Camellia	*fraterna*	8
Theaceae	Cleyera	*japonica*	3
Theaceae	Eurya	*muricata*	75
Theaceae	Eurya	*rubiginosa*	2
Theaceae	Schima	*superba*	46
Theaceae	Ternstroemia	*gymnanthera*	3

Fig. S1. Functional trait dendrogram of the species found in the experimental plots. Branch lengths reflect Euclidean distances in a normalized trait space (σ=1 along each axis). Functional diversity (FD) was calculated for each plot as total branch length of the subtree containing the species present.

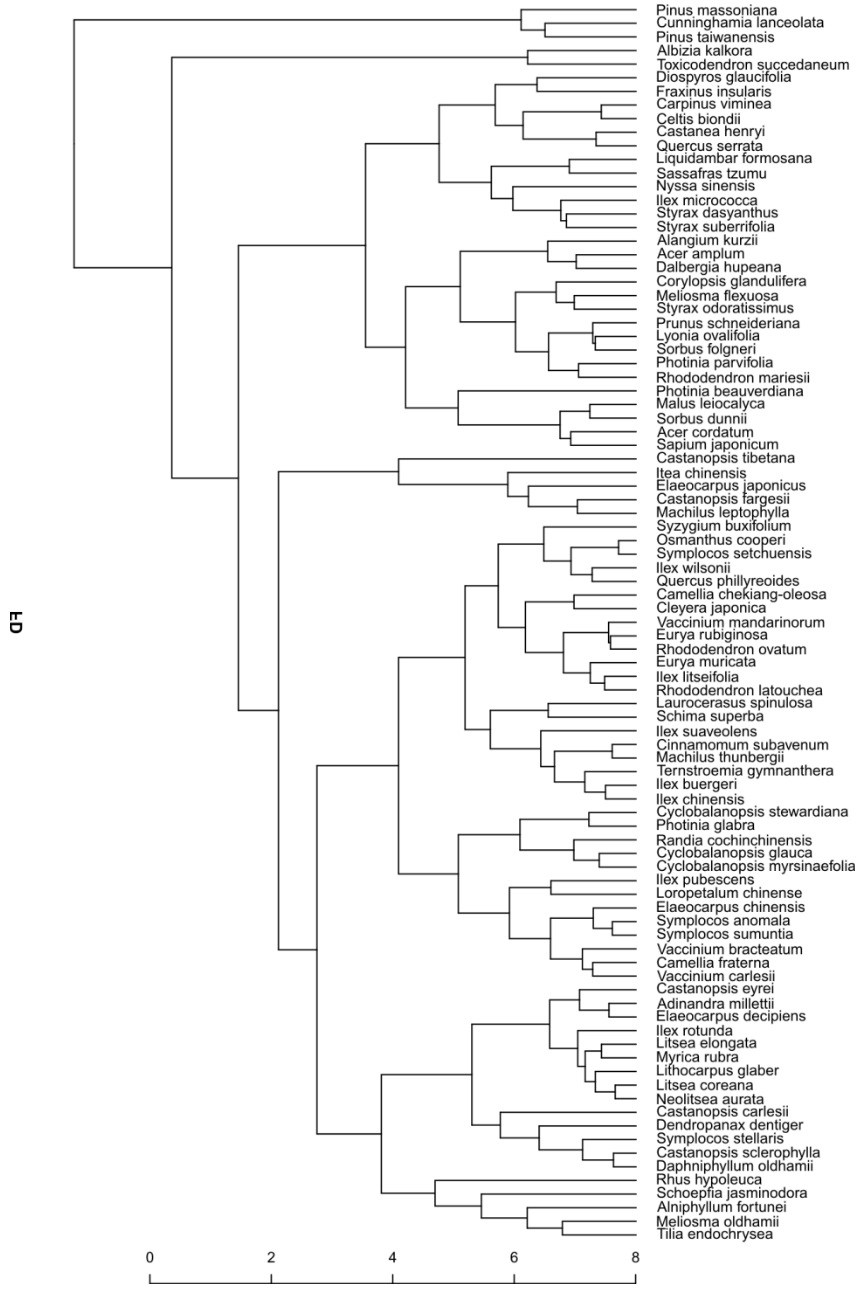

Fig. S2: Phylogeny of species found in the study plots. Phylogenetic diversity (PD) was calculated for each plot as total branch length of the subtree containing the species present.

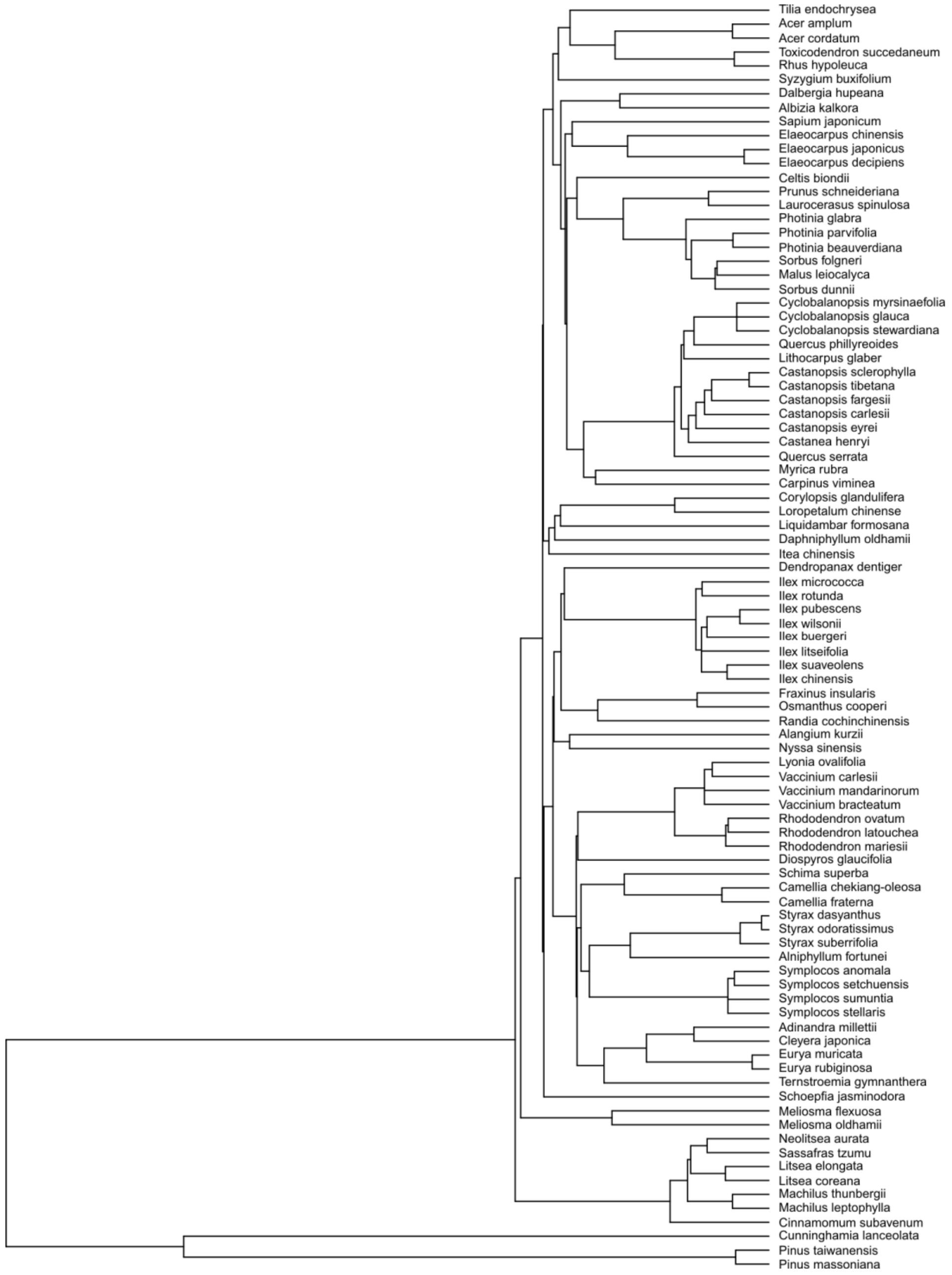

Method S1. Generation of phylogenetic tree

We gathered sequence information, i.e. *mat*K, *rbc*L and the ITS region including the 5.8s gene for all woody species from Gutianshan National Nature Reserve [1] or closely related species available in GenBank (http://www.ncbi.nlm.nih.gov/genbank/, accessed between May and June 2012). For some species found in the study plots, *mat*K and *rbc*L were sequenced using standard barcoding protocols [2] (Table S3, accessions KF569888-KF569890). All sequences were aligned separately for the different markers using MAFFT v6 [3]. Sequences for *mat*K and *rbc*L were aligned with the 'Auto' option in the online version of the program (http://mafft.cbrc.jp/alignment/server/). The ITS region was aligned with the 'Q-INS-I' option considering secondary structure of RNA using the MAFFT application at Bioportal [https://www.bioportal.uio.no/, 4]. Aligned sequences were concatenated for each species resulting in a total alignment of 3521 nucleotide positions. A phylogenetic tree was inferred using a Maximum Likelihood (ML) method implemented in PhyML [5]. For ML inference, the best fitting model (GTR+I+G) selected by Modeltest [6] was applied with the following options: tree topology search operation: best of NNI and SPR search, number of substitution rate categories =6, all other parameters were estimated (Gamma Distribution Parameter Alpha, Proportion of Invariable Sites, Transition/Transversion Ratio).

Four species occurring in the CSPs but without sequence information available (Table 1) were added manually to the obtained ML tree by the following procedure. *Acer cordatum* was added within *Acer* as a polytomy to the most recent common ancestor (MRCA) of a monophyletic clade formed by other members of *Acer* sect. *Palmata* (i.e. *A. elegantulum*, *A. wilsonii*, *A. olivaceum*). Its branch length was defined as the average distance from the MRCA of that clade to the tips. *Styrax wuyuanensis*, *Symplocos oblongifolia* and *Vaccinium mandarinorum* were added similarly as polytomy emerging from the MRCA for all other members of the respective genus included, with branch lengths equalling the average branch length from that MRCA to the tips of congeners.

Using the ML topology and branch lengths an ultrametric tree was created by non-parametric rate smoothing (nprs) as implemented in r8s [7]. Absolute node ages were obtained using 27 published fossils or dates as age constrains. A fixed age of 125 million years was applied to the crown node of the Eudicots (Table S4).

Table S3. List of woody species in the Gutianshan National Reserve included in the phylogenetic analysis.

Species	Species substitute or synonym	GeneBank accession number		
		***mat*K**	***rbc*L**	**5.8s+ITS**
Abelia_chinensis		AY310461	HQ680737	FJ745388
Abutilon_theophrasti		HM850990	HM849734	DQ006017
Acanthopanax_trifoliatus		U58603	U50239	
Acer_amplum	*Acer campestre*	JN894032	DQ978399	DQ238431
Acer_buergerianum			DQ978396	U89908
Acer cordatum	added manually to ML tree			
Acer_davidii		JF952989	DQ978406	JF975773
Acer_elegantulum		HQ427339	HQ427191	
Acer_mono			DQ978416	AY605447
Acer_olivaceum		HQ427338		
Acer_tataricum			DQ978436	AY605363
Acer_wilsonii		HQ427337	HQ427189	HM352665
Actinidia_callosa		AF322620	AJ549061	AF323829
Actinidia_chinensis		U61324	L01882	
Actinidia_hemsleyana		AF322608	AJ549036	AF323802
Actinidia_lanceolata			AJ549072	
Actinidia_melanandra		AF322600		AF443211
Adina_rubella			AJ346965	AJ346856
Adinandra_millettii		AF380069	HQ427223	AY626848
Aesculus_chinensis		EU687709		JF421459
Ailanthus_altissima		EF489111	HM849750	JF755934
Akebia_quinata		AF542587	L12627	GQ339575
Akebia_trifoliata		GQ434168	AF335305	AY029788
Alangium_kurzii		FJ644650	DQ340449	FJ610018
Alangium_platanifolium		FJ644640	JF308649	FJ610006
Albizia_julibrissin		AY386855	GU135262	FJ572041
Albizia_kalkora		HQ427295	HQ427141	JF708202
Alniphyllum_fortunei		HQ427279	AF396149	AF396437
Amelanchier_asiatica				JQ392362
Antidesma japonicum	*Antidesma venosum*	HQ415372	JF265291	
Aphananthe_aspera		AF345320	AF500339	

Aralia_chinensis		HQ427393	HQ427250	U63181
Aralia_dasyphylla				DQ007355
Aralia_echinocaulis				AF273525
Ardisia_brevicaulis				FJ482141
Ardisia_crenata		HQ427412	L12599	JN645186
Ardisia_crispa				FJ482139
Ardisia_hanceana				JN645190
Ardisia_japonica		JF416274	GQ436756	JN645201
Berberis soulieana	*Berberis fortunei*		FJ449857	FJ980428
Berchermia huana	*Berchemia zeyheri*	JF270656	JF265303	
Betula_luminifera		FJ011821		AY761116
Bischofia polycarpa	*Bischofia javanica*	GU135116	AY663571	
Broussonetia_papyrifera		AF345326	JF317478	HM623778
Buddleja lindeyana	*Buddleja davidii*	HQ384530	AJ001757	
Buxus sinica	*Buxus sempervirens*	AF543728	HM849831	EF123195
Caesalpinia_decapetala		HM049555		JF708207
Callicarpa_bodinieri		HQ427330	HQ427182	
Callicarpa_giraldii		HQ427332	HQ427184	FJ593347
Callicarpa_japonica		FM163257		FM163230
Callicarpa_rubella		HQ427329	HQ427181	FM163232
Camellia_brevistyla				HM061465
Camellia_chekiangoleosa		HQ427374	HQ427229	EU579685
Camellia_cuspidata		HQ427370	HQ427225	EU579693
Camellia_fraterna			HQ427224	EU579705
Camellia_oleifera			GQ436647	HM061454
Camellia_sinensis		AJ429305	AF380037	HM061514
Camptotheca_acuminata		JF953409	L11211	JF976064
Campylotropis_macrocarpa		AY386870	EU717277	GU572164
Caragana_sinica		HM049541	FJ537233	FJ537284
Carpinus_londoniana		AY211990		AF432040
Carpinus_viminea		AY212000	HQ427161	AF432058
Castanea_henryi		EF057123		
Castanea_mollissima		EF057124		
Castanea_seguinii		AY263920	AY263937	
Castanopsis_carlesii		AY040496	HQ427175	AY040372

Castanopsis_eyrei		EF057125	HQ427167	EF057109
Castanopsis_fargesii		EF057133	HQ427173	AY040383
Castanopsis_sclerophylla		EF057137		EF057106
Castanopsis_tibetana		AY263921	AY147096	
Celastrus_aculeatus				JQ424095
Celastrus_angulatus		EU328938		JQ424098
Celastrus_gemmatus				JQ424102
Celastrus_oblanceifolius				JQ424119
Celastrus_rosthornianus		EU328940		JQ424130
Celastrus_stylosus				JQ424136
Celtis_biondii		KF569895	KF569888	
Celtis_tetrandra			JF317479	
Cephalotaxus_fortunei		AF228109	AY450863	
Cephalotaxus_sinensis		AF228110	EF660728	
Cerasus_campanulata	syn. *Prunus campanulata*		AF411501	AF318658
Chimonanthus_salicifolius		HQ427325	HQ427177	AY786102
Choerospondias_axillaris		HQ427341	HQ427193	GQ434625
Cinnamomum_camphora		AJ247154	L12641	AY878325
Cinnamomum_chekiangense		HQ427409	HQ427267	
Cinnamomum_subavenium		HQ427408	HQ427266	GU598529
Cladrastis wilsonii	*Cladrastis sikokiana*		U74232	JQ676968
Clerodendrum_bungei				U77744
Clerodendrum_cyrtophyllum		HQ427333	HQ427185	JF755940
Clerodendrum_trichotomum		AF477760	HQ427186	U77771
Clethra_barbinervis		AB697681	AF421089	AY190573
Cleyera_japonica		HQ427371	EU980811	AF456257
Coptosapelta_diffusa			EU145453	DQ358882
Corchoropsis_crenata			FJ848895	
Corchorus_aestuans				DQ311675
Cornus_controversa		U96893	AF190433	AY530918
Cornus_kousa		DQ341345	L14395	DQ340555
Corylopsis_glandulifera	syn. *Corylopsis hypoglauca*	HQ427314	HQ427165	EF456719
Corylopsis_sinensis		AF013038	AB237032	EF456711
Crataegus cuneata	*Crataegus monogyna*	JN893932	JN890652	
Cryptomeria_fortunei		AB030117		

Cunninghamia_lanceolata		AB030125	AY140260	
Cyclobalanopsis_glauca	syn. *Quercus glauca*	AB060062	AB060571	AY040458
Cyclobalanopsis_gracilis	syn. *Quercus ciliaris*	HQ427318	HQ427169	
Cyclobalanopsis_nubium	syn. *Quercus sessilifolia*	AB060068	AB060577	
Cyclobalanopsis_stewardiana		KF569896	KF569889	
Cyclocarya_paliurus		AY147098	AY147094	AF303817
Dalbergia_hupeana		HQ427296	U74236	GU217673
Daphne genkwa	*Daphne laureola*	JN894952	HM849946	GQ167533
Daphniphyllum_macropodum			AM183400	
Daphniphyllum_oldhamii		HQ427311	HQ427162	JN040993
Dendropanax_dentiger		HQ427394	HQ427251	GU054694
Deutzia glauca	*Deutzia setchuenensis*	JF308687	JF308658	
Diospyros_glaucifolia		HQ427382	EU980694	FJ624405
Diospyros_morrisiana		HQ427383	HQ427240	
Diospyros_oleifera		AB174997		AB175016
Diospyros_rhombifolia		AB174999	EU980741	AB175018
Distylium_myricoides		GU576683	AM183408	GU576648
Edgeworthia_chrysantha			AJ297920	AJ744932
Ehretia_thyrsiflora			EU599831	
Elaeagnus_glabra				JQ062502
Elaeagnus_multiflora				JQ062478
Elaeagnus_pungens		GU135102	GU135269	JQ062488
Elaeagnus_umbellata		AY257529	HM849968	JQ062486
Elaeocarpus_chinensis			HQ427153	
Elaeocarpus_decipiens		HQ415261	HQ415077	
Elaeocarpus_japonicus		HQ415264	HQ415080	
Eleutherococcus_gracilistylus			GQ436710	FJ980422
Emmenopterys_henryi		FJ905360	Y18715	FJ984985
Euchresta_japonica			AB127040	
Euodia faugeaii	*Euodia hupehensis*	EF489105	FN552679	
Euonymus_alatus		EU328950		EU328755
Euonymus_carnosus		HQ427389	HQ427246	
Euonymus_centidens		HQ427390	HQ427247	
Euonymus_fortunei		HQ393828	HM755927	HQ393699
Euonymus_myrianthus		HQ427388	HQ427245	HQ393721

Euonymus oblongifolius	syn. *Euonymus nitidus*	HQ393835	HQ427248	JQ424144
Euonymus_oxyphyllus		HQ393836		HQ393704
Eurya_alata				AF456259
Eurya_hebeclados				AY626865
Eurya_loquaiana		HQ427372	HQ427227	AY626870
Eurya_muricata		HQ427373	HQ427228	AY626872
Eurya_nitida				AY096026
Eurya_rubiginosa		HQ427368	HQ427222	AY626877
Euscaphis_japonica		DQ663628	DQ307099	
Fagus_engleriana		AY042391	JF941501	AY232907
Fagus_longipetiolata		AY042402	JF941508	AY232955
Fagus_lucida		EF057139	JF941510	AY232963
Ficus_erecta		HQ427366	HQ427220	HQ890729
Ficus_heteromorpha			JF941536	
Ficus_pandurata		HQ415327	HQ415153	
Ficus pumila		HM851109	AF500352	AY063580
Ficus_sarmentosa				AB485901
Firmiana_platanifolia			AY328192	AF460185
Fontanesia fortunei	syn. *Fontanesia phillyreoides*			AF534815
Forsythia_viridissima		FJ263957		AF534810
Fraxinus_chinensis		HM171509	DQ673301	HQ705225
Fraxinus_insularis		HQ427335	HQ427187	
Gardenia_jasminoides		HQ427344	GQ436564	GQ434646
Gardneria_multiflora				JF937929
Gleditsia_sinensis		AM086835		AF510019
Glochidion_puberum		HQ427285	AY663586	AY936659
Gymnocladus_chinensis				AF510033
Hamamelis_mollis		AF128827	L01922	GU576659
Helwingia_japonica		AJ430195	L11226	AF200593
Hibiscus_syriacus		AF345329	AY328174	AF460188
Holboellia coriacea	*Holboellia grandiflora*	FJ626513	AF398181	AY029779
Hovenia_dulcis				DQ146607
Hovenia_trichocarpa		JF317429	JF317489	DQ146608
Hydrangea_angustipetala		GU217336		
Hydrangea_anomala		GU369710	AF323202	JF976651

Hydrangea_chinensis		KF569897	KF569890	AB377211
Hydrangea_paniculata		HQ427310	AB236036	
Hydrangea strigosa	syn. *Hydrangea aspera*	AJ429277	JF941958	JF976653
Idesia_polycarpa		FJ670040	AF206781	AJ006441
Ilex_buergeri			FJ394593	FJ394663
Ilex_cornuta		GQ997309	FJ394601	EU647650
Ilex_elmerrilliana			HQ427132	
Ilex_ficoidea		HQ427288	HQ427133	FJ394682
Ilex_latifolia		HQ427289	X98731	DQ200798
Ilex_litseaefolia		KF569898		
Ilex_macrocarpa			AJ4927271	AJ492689
Ilex_micrococca		HQ427290	X98721	JF976691
Ilex_pubescens		HQ427291	AJ492722	AJ492686
Ilex_purpurea		HQ427292	AJ492710	FJ394708
Ilex_rotunda		HQ415255	X98720	FJ394710
Ilex_suaveolens		HQ427293	HQ427139	
Ilex_triflora			AJ4927131	AJ492675
Ilex_tsoii			FJ394645	FJ394718
Ilex_wilsonii		HQ427294	FJ394649	FJ394722
Illicium_lanceolatum		HQ427283	HQ427126	JQ180205
Indigofera_decora				AF534797
Itea_chinensis		HQ415356	HQ415186	
Jasminum sinsense	*Jasminum nudiflorum*	AF531779		AF361301
Juglans_cathayensis		AF118028		
Juniperus_chinensis		HM024014	HM024292	
Juniperus_formosana		HM024028	HM024306	
Kerria_japonica		AB073686	AF132893	
Koelreuteria_bipinnata			DQ978447	
Lasianthus_japonicus		HQ427345	HQ427196	
Lespedeza_buergeri				JN402408
Lespedeza_cyrtobotrya				JN402422
Lespedeza_dunnii				JN402431
Lespedeza_floribunda		HM049538	GQ436353	JN402438
Lespedeza_formosa	syn. *Lespedeza thunbergii*		HQ427143	JN402486
Ligustrum_lucidum		EU669873	GQ436542	JF976848

Ligustrum_sinense	JF830514	JF830433	JF830366
Lindera_aggregata	AB442057	HM019473	AB470487
Lindera_erythrocarpa	AB259065		HQ697215
Lindera_glauca	AB442056	HM019478	AB500615
Lindera_megaphylla	AF244404		AY265406
Lindera_reflexa	AF244401	HQ427264	AY265407
Liquidambar_acalycina	AF015649	DQ352380	GU576668
Liquidambar_formosana	AF133221	AJ131772	AF015436
Liriodendron_chinense	AF123481	AY841593	
Lithocarpus_cleistocarpus	EF057117		EF057114
Lithocarpus_glaber	HQ427322	AB060568	AY040435
Lithocarpus_hancei			AY040451
Litsea_coreana	HQ427405	HQ427263	AF272286
Litsea_cubeba	AF244398	AY337734	AB260863
Litsea_elongata	HQ427403	HQ427261	DQ120606
Lonicera_hypoglauca	HM228434	HM228478	FJ372916
Lonicera_japonica	GQ997392	HM850134	JQ780992
Lonicera_macranthoides	HM228448	HM228492	FJ372918
Lonicera_modesta			EU240716
Loropetalum_chinense	HQ427312	AF061999	GU576672
Lyonia_ovalifolia	U61305	AF124580	
Maackia_chinensis			EF457721
Machilus_grijsii	KF569899	KF569893	JF976985
Machilus_leptophylla	HM019350	HM019490	EF538697
Machilus_pauhoi	HQ427418	HM019496	EF538695
Machilus_thunbergii	KF569890	KF569894	FJ755429
Maesa_japonica			JF708192
Magnolia_cylindrica	HQ427420	AY008914	
Magnolia_denudata	AF123465	AY008913	EU593545
Magnolia_officinalis	AF548641	AY008933	EU593549
Mahonia_bealei	DQ478617	L12657	FJ424229
Mallotus_japonicus	AB268027	AY794934	
Mallotus_repandus	EF582678	GU441787	DQ866617
Malus_hupehensis	AF309179	JQ391346	JQ392455
Malus_leiocalyca	HQ427351	HQ427202	

Manglietia_fordiana		AY952412	L12658	
Melastoma_dodecandrum			GQ436727	GQ265883
Melia_azedarach		EF489117	AY128234	AY695595
Meliosma_flexuosa		HQ427361	HQ427214	
Meliosma_oldhamii		HQ427360	HQ427213	
Meliosma_rigida		HQ415309	HQ415132	
Michelia_maudiae		HQ415276	HQ415093	EU593553
Michelia_skinneriana		HQ427417	HQ427275	
Microtropis_fokienensis		HQ393848		HQ393683
Millettia dielsiana	syn. *Callerya cinerea*		GQ436360	FJ980295
Millettia reticulata	syn. *Callerya reticulata*	AF142733		AF467031
Morus_alba		AB038183	L01933	JN407493
Morus_australis		GU145559	GU145573	AY345152
Morus_cathayana		GU145565	GU145579	AM042001
Mussaenda_shikokiana				AJ846854
Myrica_rubra	syn. *Morella rubra*	HQ427396	HQ427253	AJ626784
Myrsine_retusa		HM850887	HM850193	
Neolitsea_aurata		HM019358	HM019498	JF977135
Nyssa_sinensis		JF308675	JF308651	EU734444
Orixa_japonica		EF489106		HM851496
Ormosia_henryi		HM049514		
Osbeckia_chinensis			AF215525	
Osmanthus_cooperi		EU669875	HQ427188	EF362772
Osmanthus_fragrans		FM208253		EU314904
Osmanthus_matsumuranus		EU409435		EF362770
Persea_grijsii		AJ247180		
Pertusadina_hainanensis		HQ427346	AJ347002	AJ346892
Philadelphus brachybotrys	*Philadelphus pekinensis*	GU217268		
Phoebe_bournei		HM019369	HM019509	EF538706
Phoebe_sheareri		HQ427400	HM019513	FM957848
Photinia_beauverdiana		HQ427353	HQ427204	JQ392492
Photinia_glabra		HQ427354	HQ427205	FJ796905
Photinia_parvifolia		HQ427355	HQ427206	GQ368497
Photinia serrulata	syn. *Photinia serratifolia*	AF288111	GQ436594	GQ368486
Photinia_villosa				FJ810016

Phyllanthus_glaucus			AY765271	HM106990
Phyllanthus_urinaria			AY765268	AY936735
Picrasma_quassioides		HQ427327	EU043008	GQ434548
Pieris_formosa		U61303	AF124581	EU547690
Pieris_japonica		AB206598	AB206589	EU547692
Pieris_taiwanensis		AB206599	AB206593	
Pinus_massoniana		DQ353716	DQ353732	
Pinus_taiwanensis		AB161016	DQ156493	
Pistacia_chinensis			FN599457	EF193079
Pittosporum_illicioides		HQ427307	HQ427157	
Platycarya_strobilacea		HQ427308	AY263933	AF303808
Pleioblastus_amarus	*Arundinaria tecta*	EF125165	AJ746179	HQ292267
Podocarpus_macrophyllus		AF228111	AF249616	
Podocarpus nagi	syn. *Nageia nagi*	AB644449	AB644468	
Polygala_arillata			AM234210	
Populus adenopoda	*Populus tremula*	AJ506086	AJ418827	
Pourthiaea_hirsuta				GQ368494
Premna_microphylla		HQ427331	U28883	
Prunus_discoidea			HQ427208	
Prunus_mume		JF955822	AF411491	JF978116
Prunus_persica		AF288117	AF411493	JF978127
Prunus_phaeosticta		HQ415272	HQ415089	EU669095
Prunus_salicina			AF411494	AF318725
Prunus_schneideriana		HQ427356	HQ427209	EU370928
Prunus_serrulata		GU363780	AF411487	AF318721
Prunus_spinulosa		HQ427357	AF411503	AF411513
Prunus_undulata				EU669108
Pseudolarix_kaempferi		AB019866	X58782	
Pterocarya insignis	syn. *Pterocarya macroptera*			AF303814
Pterocarya_stenoptera		AF118042		AF179587
Pyrus_calleryana			JQ391379	JQ392478
Quercus_acutissima		AB060069	AB060578	AF098428
Quercus_fabri				HE591366
Quercus_myrsinifolia		AB060063	AB060572	AF098414
Quercus_phillyraeoides		HQ427324	AB060573	AY040462

Quercus_serrata		AB060067	AB060576	
Quercus_variabilis		AB060065	AB060574	AY040463
Randia_cochinchinensis	syn. *Aidia cochinchinensis*	HQ427347	HQ427198	
Rhamnella_franguloides			AJ3900271	AY626454
Rhamnus_crenata		HQ427385	HQ427242	AY626443
Rhamnus_utilis		JF317432	JF317492	
Rhaphiolepis_indica		HQ427352	HQ427203	JQ392494
Rhododendron_fortunei		AF454850	HQ706905	AF393407
Rhododendron_latoucheae		HQ427298	HQ427145	
Rhododendron_mariesii		AF454860	HQ427147	AF297202
Rhododendron_ovatum		U61330	HQ427144	JF978354
Rhododendron_simiarum			HQ706935	HQ707070
Rhododendron_simsii		HQ427299	GQ997829	JF978401
Rhus_chinensis			FN599458	EF682845
Rhus_hypoleuca		HQ427342		
Rosa_bracteata		HM490026		
Rosa_cymosa		AB039317		HM593924
Rosa_henryi		AB039310		AB038454
Rosa_laevigata		AB011997	GU363797	JN407516
Rosa_multiflora		AB039304	GQ436573	HM593923
Rosa_rubus		FJ472525		FJ416660
Rubus_amphidasys				AY083367
Rubus_buergeri				FJ472903
Rubus_chingii		HQ427358	HQ427211	
Rubus_corchorifolius				JF708203
Rubus_coreanus				FJ472906
Rubus_hirsutus		GU363753	GU363792	FJ472891
Rubus_hunanensis				FJ472902
Rubus_irenaeus				EF034131
Rubus_lambertianus				FJ472904
Rubus_parvifolius		AB073699	GU363802	JN407526
Rubus_pungens				FJ472893
Rubus_reflexus		JN407197	JN407362	JN407520
Rubus_swinhoei				EF034143
Rubus_tephrodes				EF034144

Rubus_trianthus				AY083366
Sabia_campanulata			AM183414	
Sabia_japonica		AM396512		
Sabia_swinhoei		GU266603	FJ626616	
Sageretia_thea			AJ2257851	AY626453
Salix_babylonica		AJ849593	FJ788588	
Sambucus_chinensis		JF956210	JF944185	JF978521
Sambucus_williamsii				JN040994
Sapindus_mukorossi			FN599461	
Sapium discolor	syn. *Triadica cochinchinensis*	HQ415366	HQ415199	JF733770
Sapium japonica	syn. *Neoshirakia japonica*		AY794856	
Sapium sebifera	syn. *Triadica sebifera*	GU135113	AY794859	GU441830
Sassafras_tzumu		AF244391	HM019516	GU082375
Schima_superba		AJ429306	Z80208	HM100443
Schoepfia_jasminodora		HQ415321	HQ415146	
Securinega suffruticosa	*Securinega capuronii*		AY663621	
Serissa foetida	syn. *Serissa serissoides*		Z68822	FJ980385
Skimmia_reevesiana		FN668822	FN599464	
Sloanea_sinensis			HQ427152	
Sorbus alnifolia	syn. *Aria alnifolia*	DQ860451		FJ810006
Sorbus_dunnii	syn. *Aria dunnii*			GQ368505
Sorbus_folgneri		HQ427359	HQ427212	
Sorbus_hemsleyi				FJ810010
Spiraea_blumei		JQ041791		JQ041773
Spiraea_cantoniensis		AF288127		DQ897609
Spiraea_chinensis		JQ041792		JQ041774
Spiraea_japonica				DQ897617
Spiraea_prunifolia		JQ041787		DQ897623
Spiraea_vanhouttei			L11206	U16205
Stachyurus_chinensis		AM396501	JF944501	DQ307102
Stauntonia_hexaphylla		FJ626517	L37922	AY029784
Stephanandra_chinensis		AF288128		AF487153
Stewartia_sinensis		AF380106	AF380061	AY070322
Styrax_calvescens				AF327468
Styrax_dasyanthus		HQ427280	HQ427123	AF327469

Styrax_faberi				AF327484
Styrax_japonicus				AF327465
Styrax_odoratissimus		HQ427282	HQ427125	AF327460
Styrax_suberifolius		HQ427281	HQ427124	AF327493
Styrax wuyuanensis	added manually to ML tree			
Symplocos_anomala		AY679808	HQ427233	AY336291
Symplocos_chinensis		AY336341		AF396229
Symplocos_heishanensis				AY630642
Symplocos_lancifolia		HQ415339	HQ415167	AB114887
Symplocos_laurina		AY336368		AY336318
Symplocos oblongifolia	added manually to ML tree			
Symplocos_paniculata		AF440433	Z83139	AY336263
Symplocos_phyllocalyx		AY336357		AY336293
Symplocos_setchuensis		AY336359	HQ427235	AY336294
Symplocos_stellaris		HQ427379	HQ427236	AY336329
Symplocos_sumuntia		HQ427377		AY336322
Syzygium_buxifolium		HQ415314	HQ427244	EF026624
Tarenna_mollissima		HQ415401		
Taxodium_distichum		JQ512482	AF119185	
Taxus_chinensis			AY450856	
Taxus_wallichiana		HM590991	HM591042	
Ternstroemia_gymnanthera		AF380109	AF421106	HM061522
Tilia_endochrysea		HQ427306	HQ427156	
Toona_ciliata				FJ462489
Toona_sinensis		JN680343	JN654542	FJ462490
Torreya_grandis		AF228108	DQ478794	
Toxicodendron_succedaneum		HQ427343	AY510144	FJ945957
Toxicodendron_sylvestre		HQ415319	AY510145	FJ945938
Toxicodendron_trichocarpum			AY510143	FJ945927
Trachycarpus_fortunei		HQ720315	AY012460	
Trema cannabina	*Trema micrantha*	GQ982115	AF062004	AY635571
Tricalysia_dubia	*Diplospora dubia*	HQ427350	HQ427201	
Tutcheria_microcarpa		HQ427376	HQ427231	AF456277
Ulmus_parvifolia		AF345321	D86316	
Vaccinium_bracteatum		AB623177	KF569892	

Vaccinium_carlesii			KF569891	
Vaccinium japonicum	syn. *Vaccinium erythrocarpum*	AF419710		AF419781
Vacciunium mandarimorum	added manually to ML tree			
Vernicia_fordii		GU135095	GU135180	
Vernicia_montana		AB268057	AY794899	
Viburnum_dilatatum		HQ591575	HQ591719	JF979005
Viburnum_erosum		HQ427362	HQ427216	JF979007
Viburnum_fordiae		JF956802	JF944784	
Viburnum_plicatum		HQ591613	HQ591754	AY265143
Viburnum_propinquum		HQ591614	HQ591755	EF462987
Viburnum_sempervirens		HQ427363	HQ427217	HQ591976
Viburnum_setigerum		EF490251	GQ248708	HQ591977
Viburnum_sympodiale		HQ591630	HQ591770	EF462988
Vitex_negundo		AB284176	JQ322525	FM200123
Weigela_japonica		HQ427364	HQ427218	AF078716
Wikstroemia_indica		HQ415322	HQ415147	
Wikstroemia_monnula			HQ427215	
Xylosma japonica	syn. *Xylosma congesta*	AB233834	AB233938	DQ521290
Zanthoxylum_ailanthoides			FN599470	HM851475
Zanthoxylum_armatum			GQ436751	HM851465
Zanthoxylum_austrosinense				HM851488
Zanthoxylum_simulans		EF489100		HM851466
Zelkova_schneideriana		AF345328		AJ622867
Zelkova_serrata			AF206835	AJ622877

Table S4. Age constraints for nodes used to create the ultra-metric tree.

Clade	Node defined by MRCA to	Calibration type	Age [Ma]	Reference
Seed plants	*Taxodium distichum - Abutilon theophrasti*	max	385	[8]
Gymnosperms	*Pseudolarix kaempferi - Taxodium distichum*	min	318	[9]
Cupressaceae	*Cunninghamia lanceolata - Taxodium distichum*	min	90	[10]
Pinaceae	*Pseudolarix kaempferi - Pinus massoniana*	min	90	[11]
Angiosperms	*Pleioblastus amarus - Abutilon theophrasti*	max	130	[12,13]
Laurales	*Chimonanthus salicifolius - Litsea cubeba*	min	108.8	[14]
Eudicots	*Holboellia coriacea - Abutilon theophrasti*	fixed	125	[15]
Ranunculales	*Holboellia coriacea - Mahonia bealei*	min	91	[16]
Berberidaceae	*Berberis soulieana - Mahonia bealei*	min	33.9	[17]
Hamamelidaceae	*Liquidambar acalycina - Corylopsis sinensis*	min	83.5	[18,19]
Fabales	*Polygala arillata - Albizia kalkora*	min	60	[20]
Malpighiales	*Vernicia fordii - Phyllanthus urinaria*	min	89.3	[21]
Salicaceae	*Idesia polycarpa - Populus adenopoda*	min	48	[22]
Fagales	*Quercus serrata - Juglans cathayensis*	min	93.5	[23,24,25]
Juglandaceae	*Cyclocarya paliurus - Juglans cathayensis*	min	55.8	[26]
Rosaceae	*Rosa cymosa - Prunus pseudocerasus*	min	37.2	[17]
Ulmaceae	*Ulmus parvifolia - Zelkova schneideriana*	min	33.9	[17]
Rutaceae-Meliaceae	*Melia azedarach - Skimmia japonica*	min	50	[27]
Myrtales	*Melastoma dodecandrum - Syzygium buxifolium*	min	60	[28]
Ericales	*Actinidia melanandra - Ardisia crenata*	min	89.6	[29]
Actinidiaceae (stem node)	*Actinidia melanandra - Rhododendron latoucheae*	min	77.05	[30]
Cornaceae	*Alangium kurzii - Cornus kousa*	min	55.8	[17]
Nyssaceae	*Camptotheca acuminata - Nyssa sinensis*	min	33.9	[17]
Hydrangeaceae	*Deutzia glauca - Hydrangea strigosa*	min	37.2	[17]
Cornales	*Camptotheca acuminata - Cornus kousa*	min	89	[30]
Oleaceae	*Osmanthus matsumuranus - Fraxinus chinensis*	min	33.9	[17]
Dipsacales	*Viburnum sympodiale - Lonicera modesta*	min	33.9	[31]
Apiales	*Pittosporum illicioides - Dendropanax dentiger*	min	37.2	[17]

References

1. Lou LH, Li GY (1998) List of seed plants in Gutianshan.

2. Fazekas AJ, Kuzmina ML, Newmaster SG, Hollingsworth PM (2012) DNA Barcoding Methods for Land Plants In: Kress WJ, Erickson DL, editors. DNA Barcodes: Methods and protocols. New York: Humana Press. pp. 223-252.

3. Katoh K, Misawa K, Kuma Ki, Miyata T (2002) MAFFT: a novel method for rapid multiple sequence alignment based on fast Fourier transform. Nucleic Acids Research 30: 3059-3066.

4. Kumar S, Skjaeveland A, Orr R, Enger P, Ruden T, et al. (2009) AIR: A batch-oriented web program package for construction of supermatrices ready for phylogenomic analyses. BMC Bioinformatics 10: 357.

5. Guindon S, Gascuel O (2003) A Simple, Fast, and Accurate Algorithm to Estimate Large Phylogenies by Maximum Likelihood. Syst Biol 52: 696-704.

6. Posada D, Crandall KA (1998) MODELTEST: testing the model of DNA substitution. Bioinformatics 14: 817-818.

7. Sanderson MJ (1997) A nonparametric approach to estimating divergence times in the absence of rate constancy. Molecular Biology and Evolution 14: 1218-1231.

8. Gerrienne P, Meyer-Berthaud B, Fairon-Demaret M, Streel M, Steemans P (2004) Runcaria, a Middle Devonian Seed Plant Precursor. Science 306: 856-858.

9. Renner S (2009) Gymnosperms. In: Hedges SB, Kumar S, editors. The Timetree of Life. Oxford: Oxford University Press. pp. 157-160.

10. LePage BA (2003) The evolution, biogeography and palaeoecology of the Pinaceae based on fossil and extant representatives. Acta Horticulturae (ISHS) 615: 29-52.

11. Gandolfo MA, Nixon KC, Crepet WL (2001) Turonian Pinaceae of the Raritan Formation, New Jersey. Plant Systematics and Evolution 226: 187-203.

12. Hughes NF, McDougall AB, Chapman JL (1991) Exceptional new record of Cretaceous Hauterivian angiospermid pollen from Southern England. Journal of Micropalaeontology 10: 75-82.

13. Hughes NF, McDougall AB (1987) Records of angiospermid pollen entry into the english early cretaceous succession. Review of Palaeobotany and Palynology 50: 255-272.

14. Crane PR, Friis EM, Pedersen KR (1994) Palaeobotanical evidence on the early radiation of magnoliid angiosperms. Plant Systematics and Evolution Supplement 8: 51-72.

15. Hughes NF, McDougall AB (1990) Barremian-Aptian angiospermid pollen records from southern England. Review of Palaeobotany and Palynology 65: 145-151.

16. Knobloch E, Mai DH (1986) Monographie der Früchte und Samen in der Kreide von Mitteleuropa. Praha. 1-219 p.

17. Manchester SR (1999) Biogeographical relationships of North American tertiary floras. Annals of the Missouri Botanical Garden 86: 472-522.

18. Magallon-Puebla S, Herendeen PS, Endress PK (1996) *Allonia decandra*: Floral remains of the tribe *Hamamelideae* (Hamamelidaceae) from Campanian strata of southeastern USA. Plant Systematics and Evolution 202: 177-198.

19. Magallón S, Herendeen PS, Crane P (2001) *Androdecidua endressi*i gen. et sp. nov., from the Late Cretaceous of Georgia (United States): Further Floral Diversity in Hamamelidoideae (Hamamelidaceae). International Journal of Plant Sciences 162: 963-983.

20. Lavin M, Herendeen PS, Wojciechowski MF (2005) Evolutionary rates analysis of Leguminosae implicates a rapid diversification of lineages during the Tertiary. Systematic Biology 54: 575-594.

21. Crepet WL, Nixon KC (1998) Fossil Clusiaceae from the Late Cretaceous (Turonian) of New Jersey and implications regarding the history of bee pollination. American Journal of Botany 85: 1122-1133.

22. Boucher LD, Manchester SR, Judd WS (2003) An extinct genus of Salicaceae based on twigs with attached flowers, fruits, and foliage from the Eocene Green River Formation of Utah and Colorado, USA. American Journal of Botany 90: 1389-1399.

23. Batten DJ (1981) Stratigraphic, palaeogeographic and evolutionary significance of late cretaceous and early tertiary normapolles pollen. Review of Palaeobotany and Palynology 35: 125-137.

24. Kedves M (1989) Evolution of the Normapolles complex. In: Crane PR, Blackmore S, editors. Evolution, Systematics, and Fossil History of the Hamamelidae, 1-7 Systematics Association Special Volume, vol 40B. Oxford: Clarendon Press.

25. Pacltová B (1966) Pollen grains of angiosperms in the Cenomanian Peruc Formation in Bohemia. Palaeobotanist 15: 52-54.

26. Crane PR, Manchester SR, Dilcher DL (1990) A preliminary survey of fossil leaves and well-preserved reproductive structures from the Sentinel Butte Formation (Paleocene) near Almont, North Dakota. Fieldiana Geology 20: 1-63.

27. Corbett SL, Manchester SR (2004) Phytogeography and fossil history of *Ailanthus* (Simaroubaceae). International Journal of Plant Sciences 165: 671-690.

28. Pigg KB, Stockey RA, Maxwell SL (1993) *Paleomyrtinaea*, a new genus of permineralized myrtaceous fruits and seeds from the Eocene of British Columbia and Paleocene of North Dakota. Canadian Journal of Botany 71: 1-9.

29. Nixon KC, Crepet WL (1993) Late Cretaceous fossil flowers of ericalean affinity. American Journal of Botany 80: 616-623.

30. Schenk JJ, Hufford L (2010) Effects of substitution models on divergence time estimates: Simulations and an empirical study of model uncertainty using Cornales. Systematic Botany 35: 578-592.

31. Manchester SR, Donoghue MJ (1995) Winged fruits of Linnaeeae (Caprifoliaceae) in the Tertiary of Western North America: *Diplodipelta* gen. nov. International Journal of Plant Sciences 156: 709-722.

Fig. S3. Structural equation models fitting total stem basal area in 2008 (a, c) and the increment of total stem basal area from 2008–2010 (b, d) in dependence of successional age, tree diversity, and tree stem density. In contrast to the path diagrams presented in Fig. 2, these models explore the possibility that variations in tree density occur independently of diversity and successional age. In particular, they do not allow for density-mediated effects of tree diversity on stand-level growth. In the light that studies controlling initial density have shown that this pathway can dominate system-level responses, the true diversity effects may be underestimated in this approach. Nevertheless, size-mediated effects of diversity remained strong and significant for stand-level basal area growth, and marginally significant for stand-level basal area of the canopy tree cohort. The diagrams show standardized path coefficients (red: positive; blue: negative) and associated statistical significances (*** $P<0.001$; ** $P<0.01$; * $P<0.05$; (*) $P<0.1$). Variable abbreviations: S = species richness, PD = phylogenetic diversity, FD = functional diversity, DIV = diversity (latent variable related to previous three), AGE = successional age, DENS = tree density, BA = total stem basal area, ΔBA = increment of total stem basal area.

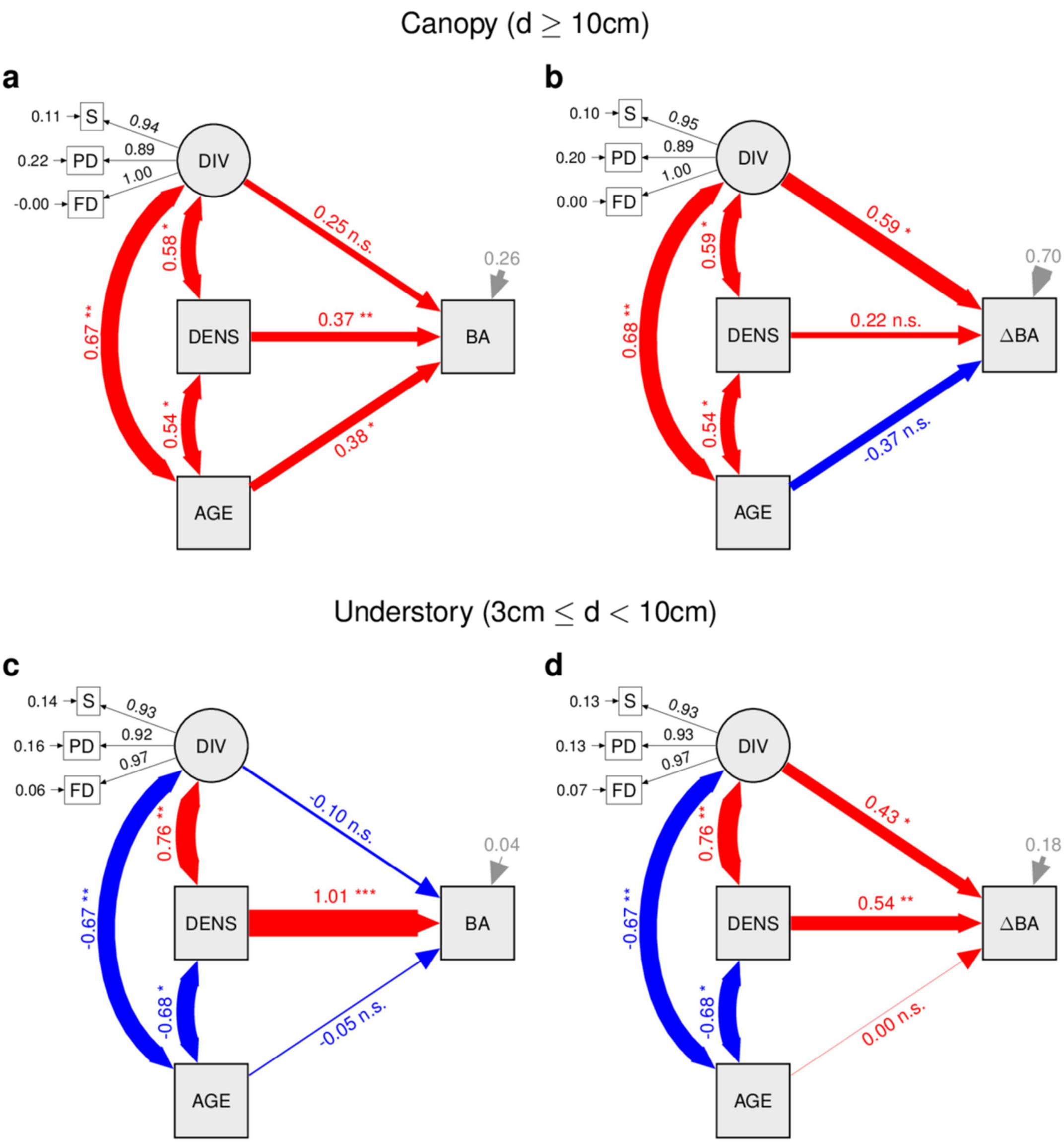
Canopy (d ≥ 10cm)
a
0.11 S
0.22 PD
-0.00 FD
0.94
0.89
1.00
DIV
0.25 n.s.
0.58 *
0.67 **
DENS
0.37 **
BA
0.26
0.54 *
0.38 *
AGE
b
0.10 S
0.20 PD
0.00 FD
0.95
0.89
1.00
DIV
0.59 *
0.59 *
0.68 **
DENS
0.22 n.s.
ΔBA
0.70
0.54 *
-0.37 n.s.
AGE
Understory (3cm ≤ d < 10cm)
c
0.14 S
0.16 PD
0.06 FD
0.93
0.92
0.97
DIV
-0.10 n.s.
0.76 **
-0.67 **
DENS
1.01 ***
BA
0.04
-0.68 *
-0.05 n.s.
AGE
d
0.13 S
0.13 PD
0.07 FD
0.93
0.93
0.97
DIV
0.43 *
0.76 **
-0.67 **
DENS
0.54 **
ΔBA
0.18
-0.68 *
0.00 n.s.
AGE
